# Assessment of the Position and Height of the Mandibular Condyle in Different Skeletal Patterns Using CBCT

**DOI:** 10.3390/jcm15186991

**Published:** 2026-09-09

**Authors:** Asel Usdat Ozturk, Sebnem Ercalik Yalcinkaya

**Affiliations:** 1 Department of Maxillofacial Radiology, Faculty of Dentistry, Istanbul Atlas University, Istanbul 34403, Turkey; 2 Department of Maxillofacial Radiology, Faculty of Dentistry, Marmara University, Istanbul 34722, Turkey

**Keywords:** cone–beam CT, linear measurement, skeletal patterns, condyle position, temporomandibular joint, mandibular condyle, malocclusion

## Abstract

**Objectives**: This retrospective cone–beam computed tomography (CBCT)-based study investigated whether sagittal and vertical skeletal patterns are associated with differences in condylar morphology, spatial position, and ramus–condyle dimensions and explored the potential clinical implications of these variations in orthodontic assessment. **Methods**: CBCT images of 132 individuals meeting predefined inclusion criteria were selected from the departmental archive. Cephalograms were reconstructed and cephalometric analyses were performed. Individuals were categorized into three sagittal skeletal pattern groups and three vertical skeletal pattern groups. Condylar morphology was evaluated using the Yale classification, and condylar position was assessed according to Pullinger’s method. Condylar and ramus heights were measured using the Habets method. Statistical analyses included Chi-square tests, one-way ANOVA, ANCOVA, and the Habets asymmetry index. Intra-observer reliability was assessed using Cohen’s kappa and intraclass correlation coefficients. **Results**: Condylar height differed significantly across vertical skeletal patterns, with the highest values in hypodivergent and the lowest in hyperdivergent individuals (right *p* = 0.004, left *p* = 0.011). A significant association was found between condyle position and vertical skeletal pattern on the right side (*p* = 0.041), but not on the left side (*p* = 0.554). Individuals with a Class III sagittal skeletal pattern showed significantly greater condylar height asymmetry (Habets index) than Class I and Class II individuals; an unadjusted difference in right ramus height (*p* = 0.001) and left condyle height (*p* = 0.046) between Class III and Class II individuals did not remain significant after adjusting for the unequal sex distribution between these groups. Males showed significantly greater condylar and ramus height than females (all *p* < 0.001). No significant relationships were identified between condylar morphology or position and sagittal skeletal patterns. **Conclusions**: A possible association was observed between vertical skeletal pattern and condylar position on the right side, which requires confirmation given the number of comparisons performed and the absence of a corresponding left-side finding. The observed differences in ramus and condylar height among skeletal groups, including significantly greater condylar height asymmetry in Class III individuals and greater ramus height asymmetry in hyperdivergent individuals, suggest that mandibular structural characteristics may vary according to underlying skeletal configuration.

## 1. Introduction

Facial growth occurs through a coordinated combination of horizontal and vertical dimensional changes, influenced by the development of craniofacial sutures, alveolar processes, mandibular condyles, and tooth eruption. Although sutural growth eventually ceases, condylar development continues beyond this period. The mandibular condyle undergoes continuous remodelling in response to functional stimuli throughout childhood and adulthood, serving as the principal growth center of the mandible. Consequently, malocclusions are believed to influence condylar morphology and position, which in turn may play a critical role in the long-term stability of orthodontic and orthognathic treatments [1,2,3,4,5,6].

The temporomandibular joint (TMJ) is a highly complex articulation that plays a central role in mastication, swallowing, breathing, and speech. As the only movable joint of the skull, the mandible articulates with the cranial base through the TMJ, also referred to as the craniomandibular joint. The TMJ consists of the mandibular condylar head and the glenoid fossa of the temporal bone, which function together to facilitate smooth mandibular movement [7]. The mandibular condyle functions as a major growth center of the mandible and indirectly contributes to the establishment of intermaxillary relationships. Accordingly, it has been suggested that the morphology and position of the mandibular condyle are associated with the sagittal skeletal pattern of the individuals in the sagittal plane [1,8].

A number of studies have reported associations between sagittal skeletal pattern and condylar morphology and position. A range of diagnostic approaches, including cephalometric and cone–beam computed tomography (CBCT) images, have been used to examine these associations [9,10,11,12,13]. Cephalograms are widely used in orthodontic diagnosis and treatment planning; however, they provide only a two-dimensional representation of inherently three-dimensional structures, thereby limiting the accuracy and detail of anatomical assessment [14]. Recent advancements in CBCT technology and associated software applications have facilitated high-resolution three-dimensional imaging and model reconstruction, thereby substantially enhancing the diagnostic evaluation and treatment planning of patients with maxillofacial deformities and malocclusions [15,16].

Although previous studies have investigated the relationship between sagittal skeletal pattern and condylar characteristics (particularly in three-dimensional and side-specific evaluations), inconsistent findings continue to limit clear clinical interpretation. Therefore, this study aimed to comprehensively investigate the association between sagittal skeletal pattern and vertical skeletal patterns and mandibular condylar morphology, position, and linear dimensions using CBCT, with particular emphasis on identifying right–left dimensional differences that may indicate a predisposition toward mandibular asymmetry.

## 2. Materials and Methods

This study was approved by the Non-invasive Clinical Research Ethics Committee of Marmara University Faculty of Dentistry (protocol no: 2020-378) and was designed in accordance with the Declaration of Helsinki. Prior to acquiring images, written consent for their future use in research was obtained from all patients. Access to the data was granted exclusively to the principal investigator.

**Figure 1:**
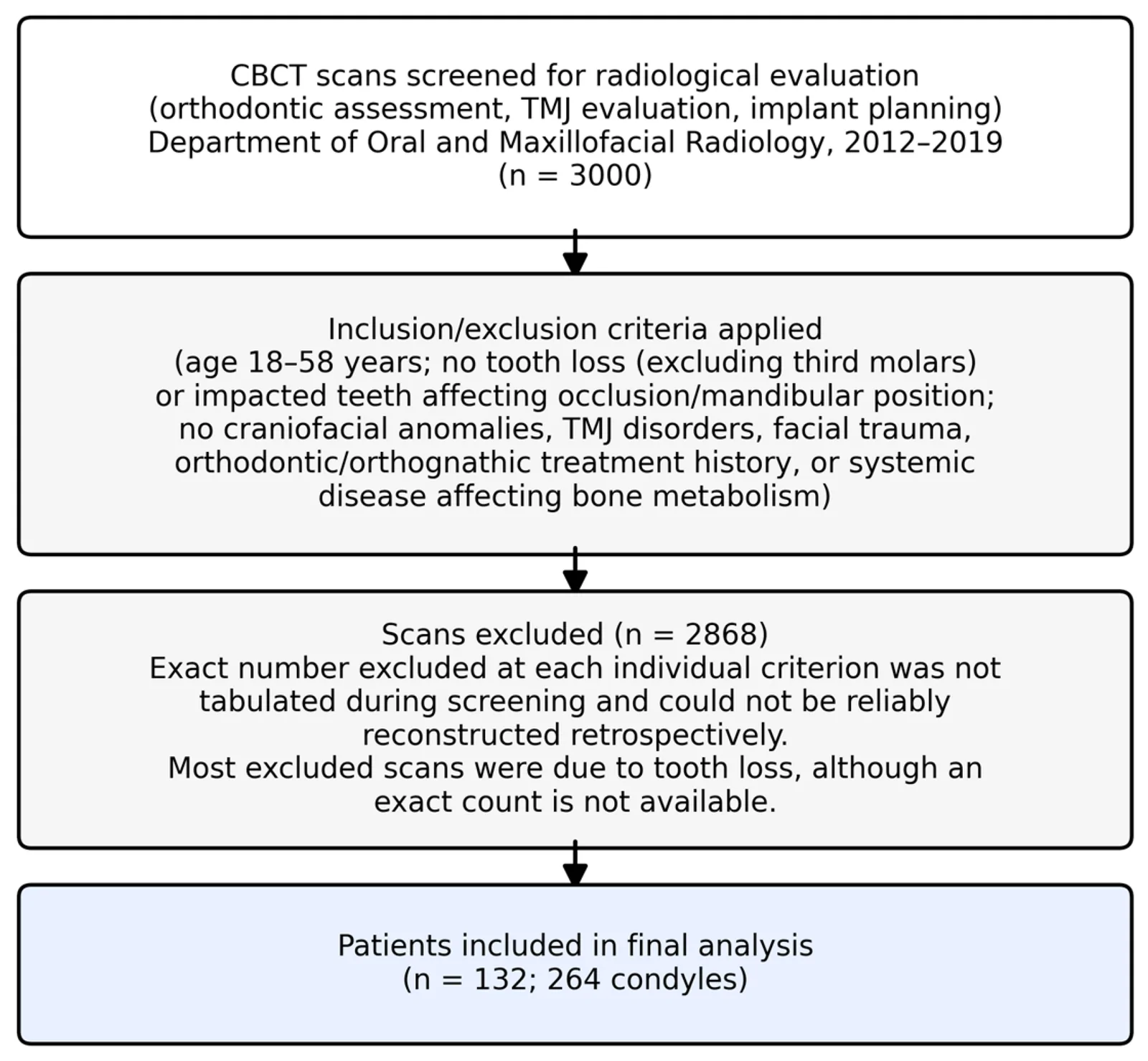
Patient selection flow diagram.

A total of 3000 CBCT images taken for radiological evaluation (including orthodontic assessment, temporomandibular joint evaluation, and implant planning) at the Department of Oral and Maxillofacial Radiology, Faculty of Dentistry, Marmara University, Istanbul, Turkey, between 2012 and 2019, were retrospectively reviewed. To eliminate potential growth-related variations, only scans of patients aged between 18 and 58 years were considered. From this dataset, 132 patients fulfilling the inclusion criteria were included in the final analysis. Eligible scans were required to be available in the institutional archive, include the anatomical regions of interest within the field of view, and demonstrate sufficient diagnostic quality for reliable evaluation. In addition, only patients without tooth loss (excluding third molars) or impacted teeth that could influence occlusion or mandibular position were included. Scans were excluded if patients had a history of facial trauma, postoperative facial deformity, orthodontic treatment (previous or ongoing), orthognathic surgery, or corrective orthopedic or functional appliance therapy. Images were also excluded in the presence of craniofacial anomalies, temporomandibular joint disorders requiring intra-articular, intramuscular, subcutaneous, or systemic medication, rheumatologic disease, maxillofacial cysts or tumors, central nervous system disorders (including encephalitis or meningitis), endocrine or metabolic diseases such as diabetes mellitus or thyroid/parathyroid disorders, or any systemic condition known to affect bone metabolism. To ensure accurate application of the eligibility criteria, patient medical histories were reviewed through both the Faculty of Dentistry database and the national health data system. Most excluded scans were due to tooth loss. The patient selection process is summarized in Figure 1.

All CBCT images were obtained using a standardized patient positioning protocol with the same Planmeca ProMax^®^ 3D Mid unit (Planmeca Oy, Helsinki, Finland), operating at 90 kVp and 10 mA, with a voxel size of 0.2 mm and a field of view (FOV) of 16 × 9 cm per acquisition. For the subset of landmarks required for full cephalometric analysis (Sella, Nasion, Porion, Orbitale, Gonion, and Menton), two sequential 360° acquisitions were obtained and merged into a single extended volume dataset via anatomical landmark-based image registration (stitching), so that the complete craniofacial region required for cephalometric tracing was captured. All scans were acquired with patients in an upright position, which may better reflect the natural condyle–fossa relationship compared with supine imaging. The images were stored in Digital Imaging and Communications in Medicine (DICOM) format and reconstructed using Planmeca^®^ Romexis^®^ software (version 2.7.0.R, Planmeca Oy, Helsinki, Finland). All analyses were performed on a 24-inch NEC MD242C2 diagnostic monitor (1920 × 1200 resolution) under standardized viewing conditions in a dimly lit room.

Prior to all measurements, CBCT images were reoriented so that the Frankfurt horizontal plane (FHP), constructed from the bilateral porion (Po) and orbitale (Or) points, was parallel to the ground. Cephalometric images were then reconstructed from the reoriented CBCT datasets. Cephalometric tracing and skeletal pattern classification were performed by an orthodontist.

Individuals were subsequently classified according to their vertical and sagittal skeletal patterns. Sagittal skeletal classification was primarily determined using the ANB angle and Wits appraisal: subjects with an ANB angle of 0–4° and a Wits appraisal between −4 and +2 mm were classified as skeletal Class I; subjects with an ANB angle >4° and/or a Wits appraisal >+2 mm were classified as skeletal Class II; and those with an ANB angle <0° and/or a Wits appraisal <−4 mm were classified as skeletal Class III [17,18,19,20,21]. Vertical skeletal pattern was evaluated using five cephalometric parameters GoMe-SN angle, maxillary height angle, Frankfort mandibular plane angle (FMA), gonial angle, and Jarabak ratio, with reference values of 25–39°, ≈60°, ≈25°, 123–137°, and 59–62%, respectively. A vertical growth pattern was assigned when at least three of the five parameters agreed: increased values (or, for the inversely scaled Jarabak ratio, decreased values) indicated a hyperdivergent pattern, decreased values (increased Jarabak ratio) indicated a hypodivergent pattern, and measurements predominantly within the reference ranges were classified as normodivergent [17,18,19,20,21,22,23].

**Figure 2:**
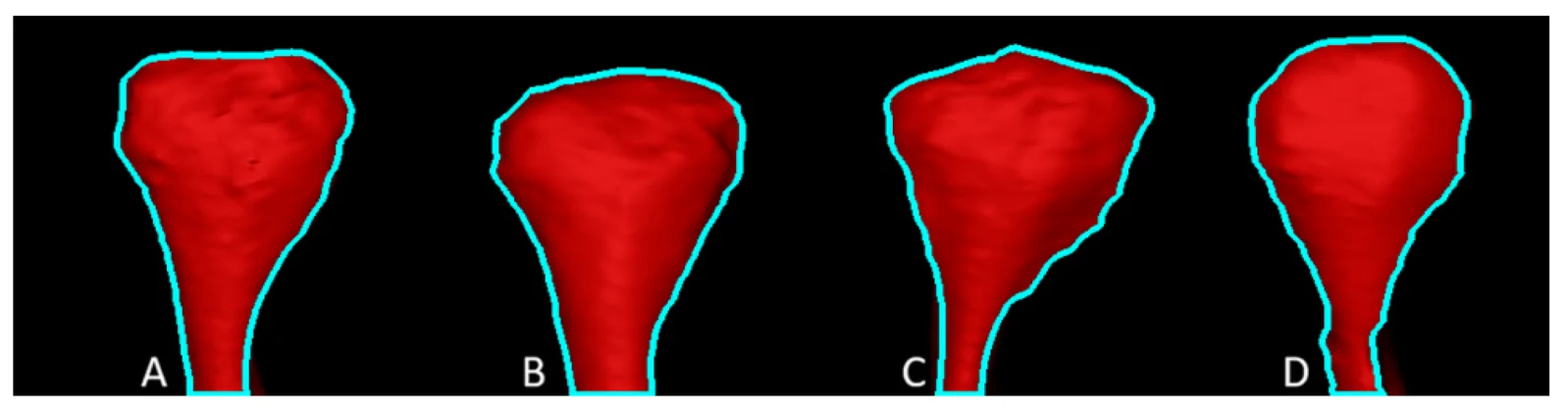
Condyle head morphology (Yale classification): (**A**): flat, (**B**): convex, (**C**): angled, (**D**): round.

**Figure 3:**
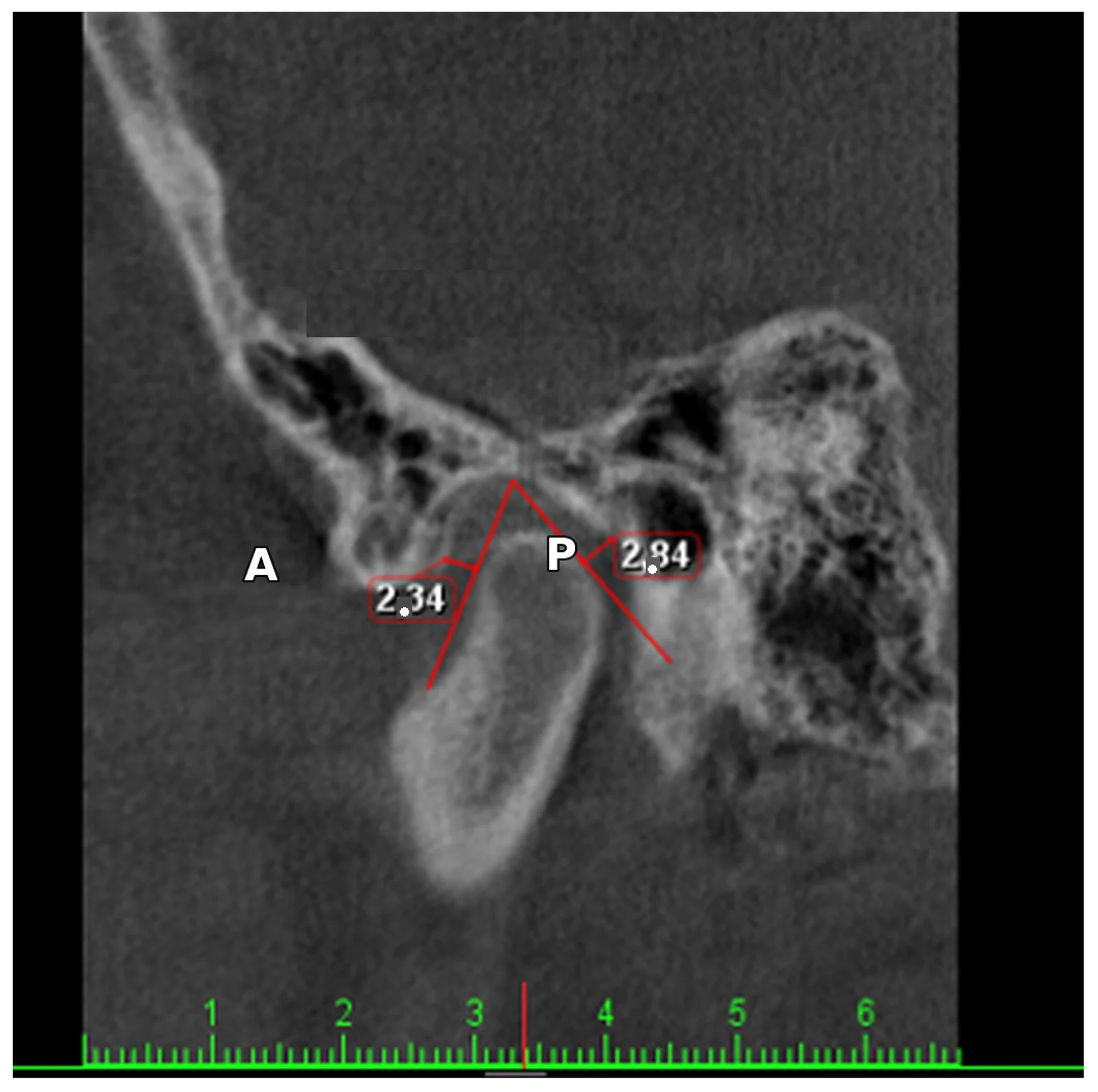
Condyle position measurement according to Pullinger’s method: CD = (A − P)/(A + P) × 100%.

**Figure 4:**
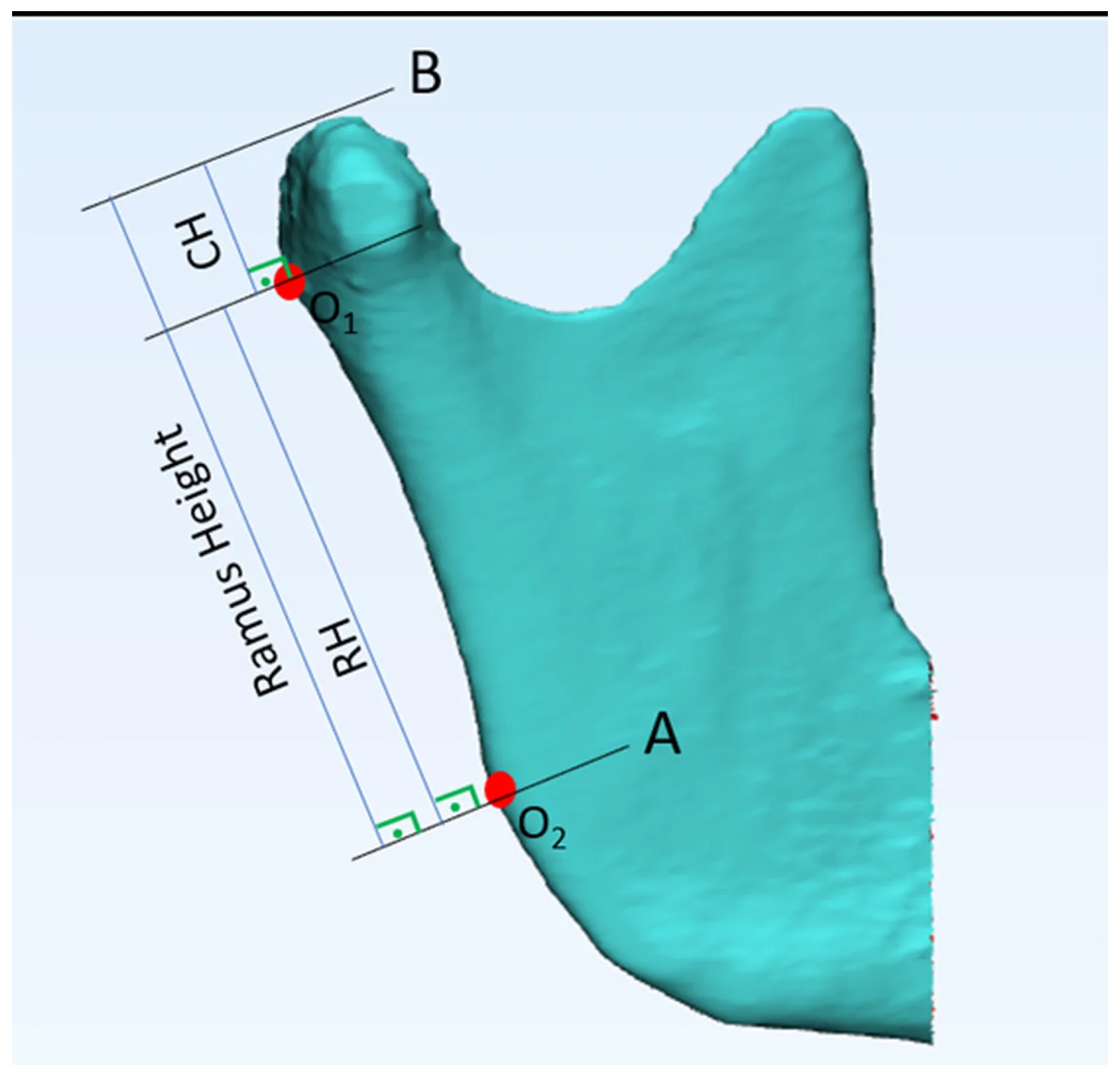
Mandibular condyle and ramus height measurements according to Habets method. O_1_: the most posterior point of the condylar head; O_2_: the most posterior point of the ramus; RH: the tangent line drawn between O_1_ and O_2_ (ramal length); B: the most superior point of the condylar head; CH: condylar height, measured as the perpendicular distance from B to the RH line; ramus height = CH + RH.

A total of 264 mandibular condyles (right and left) were evaluated. Condylar morphology in the coronal plane was classified using the Yale system as flat, convex, angled, or round [24] on 0.40 mm coronal slices, at the slice showing the maximal width of the condylar head (Figure 2). Condylar position in the sagittal plane was assessed using Pullinger’s method, on the sagittal slice where the condyle was widest and the walls of the glenoid fossa could be clearly assessed: a line drawn from the most superior point of the glenoid fossa to the condylar head was aligned with two tangents passing through the anterior and posterior aspects of the condyle, and perpendiculars from these tangents to the glenoid fossa yielded the anterior (A) and posterior (P) joint space values. Condylar displacement (CD) was then calculated as CD = (A − P)/(A + P) × 100%, with CD  ≤  −12% classifying the condyle as anterior, −12%  <  CD  <  12% as centric, and CD  ≥  12% as posterior (Figure 3) [25,26,27]. Condylar and ramus heights were measured bilaterally on sagittal sections of the three-dimensional reconstruction according to the method described by Habets et al.: a tangent line (RH) was drawn to the ramus between the most posterior point of the condylar head (O_1_) and the most posterior point of the ramus (O_2_); condylar height (CH) was measured as the perpendicular distance from the most superior point of the condylar head (B) to the RH line, and total ramus height was calculated as CH + RH (Figure 4) [28].

Statistical analyses were performed using IBM^®^ SPSS^®^ Statistics 22 (IBM Corp, Armonk, NY, USA). All measurements were conducted by a single researcher. To assess intra-observer reliability, 20 randomly selected CBCT scans were re-evaluated after four weeks. Cohen’s kappa was used to assess agreement for categorical variables, whereas the ICC was calculated to assess reliability for quantitative measurements.

The normality of the distribution of quantitative variables was assessed using the Shapiro–Wilk test. Descriptive statistics (mean, standard deviation, and frequency) were used to summarize the data. Categorical variables (condylar morphology and position) were compared using the Chi-square test. Quantitative variables (condylar and ramus height) were compared between two groups using Student’s *t*-test for normally distributed data and the Mann–Whitney U test for non-normally distributed data; associations between age and linear measurements were assessed using Spearman’s correlation coefficient. Comparisons among more than two groups were performed using one-way ANOVA for normally distributed data and the Kruskal–Wallis test for non-normally distributed data, with the Tukey HSD test used for post hoc pairwise comparisons after ANOVA and Dunn’s test with Bonferroni correction used for post hoc pairwise comparisons after the Kruskal–Wallis test, where applicable. To assess condylar and ramus asymmetry, the Habets asymmetry index was calculated for each subject as AI = |Right − Left|/[0.5 × (Right + Left)] × 100%, separately for condylar height and ramus height, and compared across sagittal and vertical skeletal pattern groups. As an empirical estimate of the measurement-error contribution to the AI, the same percentage-difference formula was applied to the two repeat measurements (four weeks apart) of the same side and subject from the intra-observer reliability subsample, providing an error floor against which the observed between-side AI values could be compared. *p* values less than 0.05 were considered statistically significant in all tests.

Effect sizes (η^2^ for ANOVA comparisons, Cramér’s V for the chi-square association) with 95% confidence intervals for the corresponding mean differences were calculated for the primary comparisons in place of a retrospective power analysis. No a priori sample size calculation was performed, as this was a retrospective analysis of an existing archival dataset. To evaluate whether a systematic right–left offset was present, paired t-tests (or Wilcoxon signed-rank tests, where the distribution of paired differences departed from normality) were used to compare right and left condylar and ramus height across the whole sample. Asymmetry index (AI) values were also compared by sex using the Mann–Whitney U test, and the sagittal-pattern comparison of condylar AI was additionally re-analyzed adjusting for sex using a rank-transformed ANCOVA, since AI was not normally distributed. Expected cell counts for all chi-square contingency tables were confirmed to be ≥5, satisfying the assumptions of the Pearson chi-square test; the Fisher–Freeman–Halton exact test was therefore not required.

## 3. Results

The age of the individuals included in the study ranged from 18 to 58 years, with 76 (57.6%) females and 56 (42.4%) males. The distribution of individuals according to sagittal skeletal pattern, vertical skeletal pattern, condylar morphology, and condylar position is presented in Table 1. When sex-related differences were evaluated, no statistically significant differences were observed in condylar morphology or condylar position between female and male individuals (Table 2).

Comparisons between the right and left sides revealed no significant differences in either condylar morphology or condylar position (*p* = 0.332 and *p* = 0.228, respectively). However, analysis of linear measurements revealed statistically significant sex-related differences in condylar and ramal dimensions. Right and left condylar heights, as well as ramus height, were all significantly greater in males than in females (*p* < 0.001) (Table 3).

No significant correlations were found between age and right condylar height (*p* = 0.211), left condylar height (*p* = 0.417), right ramus height (*p* = 0.614), or left ramus height (*p* = 0.081) (Table 3).

**Figure 5:**
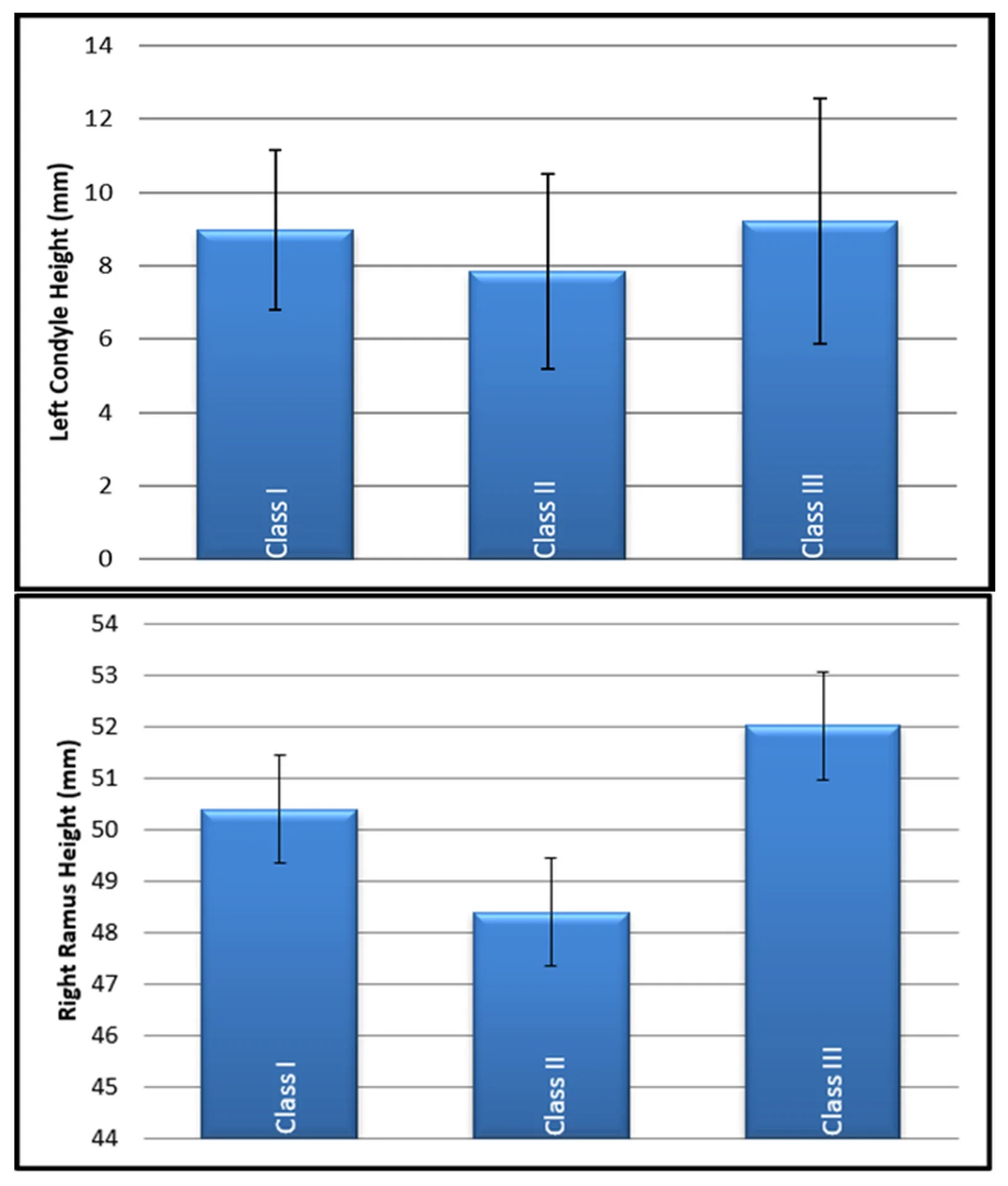
Graphic of the left condyle and right ramus in sagittal skeletal pattern.

With respect to sagittal skeletal patterns, no significant association was identified between sagittal pattern and right condylar height (*p* = 0.082). However, statistically significant differences were found in left condylar height (*p* = 0.046) and right ramus height (*p* = 0.001) among the sagittal skeletal groups. Further intergroup analysis revealed that skeletal Class III pattern individuals exhibited significantly greater right ramus height and left condylar height compared with Class II individuals (*p* = 0.001 and *p* = 0.040, respectively) (Table 4, Figure 5). The effect size for right ramus height across sagittal skeletal patterns was η^2^ = 0.096, with a mean difference of 3.64 mm (95% CI: 1.83–5.45) between Class III and Class II individuals; for left condylar height, the mean difference was 1.38 mm (95% CI: 0.06–2.70). Because the sex distribution differed markedly between the sagittal skeletal pattern groups (Class III: 25/38 male; Class II: 11/44 male; Class I: 20/50 male), and sex was independently associated with both ramus and condylar height, these sagittal pattern comparisons were re-analyzed with sex as a covariate (ANCOVA). After adjustment for sex, the sagittal-pattern effect was no longer statistically significant for either right ramus height (*p* = 0.080) or left condylar height (*p* = 0.314), indicating that the unadjusted Class III versus Class II differences reported above may be substantially attributable to the unequal sex distribution between these groups rather than to the sagittal skeletal pattern itself.

The Habets asymmetry index (AI) for condylar height differed significantly among sagittal skeletal pattern groups (Kruskal–Wallis, *p* = 0.004), with the highest asymmetry in Class III (22.7% ± 13.7%), followed by Class II (17.5% ± 16.7%) and Class I (14.7% ± 13.3%). Post hoc pairwise comparisons (Dunn’s test, Bonferroni-corrected) showed that Class III individuals had significantly greater condylar height asymmetry than both Class I (*p* = 0.004) and Class II (*p* = 0.038) individuals, while Class I and Class II did not differ significantly from each other (*p* = 1.000). The AI for ramus height did not differ significantly among sagittal skeletal pattern groups (*p* = 0.201). With respect to vertical skeletal pattern, the AI for ramus height differed significantly (*p* = 0.026), with hyperdivergent individuals showing greater asymmetry (7.4% ± 5.8%) than hypodivergent individuals (4.4% ± 4.2%; Dunn’s test *p* = 0.026), while the AI for condylar height did not differ significantly by vertical pattern (*p* = 0.231). The empirical measurement-error floor for this percentage-difference calculation, estimated from repeat measurements of the same side four weeks apart, was low relative to the between-side AI values (mean 3.9%, median 2.5%, combined across sides), indicating that measurement imprecision alone is unlikely to account for the magnitude of the observed condylar height AI or for the differences between sagittal skeletal pattern groups, since this error floor does not vary systematically between groups.

A paired comparison of right and left values across the whole sample showed no significant systematic offset for condylar height (mean difference 0.08 mm; paired *t*-test *p* = 0.646), but a small, statistically significant right-greater-than-left offset for ramus height (mean difference 0.88 mm; Wilcoxon signed-rank *p* = 0.005). To assess whether this ramus offset could account for the vertical-pattern difference in ramus AI, the analysis was repeated after centering the right and left ramus values on this offset; the hyperdivergent-versus-hypodivergent difference remained significant and, if anything, was slightly stronger (Kruskal–Wallis *p* = 0.006; Dunn’s test *p* = 0.004), indicating that this finding is not attributable to the systematic offset alone. AI values did not differ significantly by sex for either condylar height (Mann–Whitney *p* = 0.122) or ramus height (*p* = 0.987), and the sagittal-pattern difference in condylar AI remained significant after adjusting for sex using a rank-transformed ANCOVA (*p* = 0.010).

An evaluation of vertical skeletal patterns revealed significant differences in condylar height measurements. There was a gradual decrease in both right and left condylar heights across the hypodivergent, normodivergent, and hyperdivergent groups. The highest condylar heights were found in hypodivergent individuals, followed by normodivergent individuals and then hyperdivergent individuals. These differences were statistically significant (*p* = 0.004 for the right condyle and *p* = 0.011 for the left condyle) (Table 4). Post hoc pairwise comparisons (Dunn’s test, Bonferroni-corrected) showed that this difference was significant specifically between hypodivergent and hyperdivergent individuals (right condyle, *p* = 0.004; left condyle, *p* = 0.012), while neither differed significantly from normodivergent individuals (all *p* > 0.10). The effect size was η^2^ = 0.106 (right side) and η^2^ = 0.081 (left side); the mean difference between hypodivergent and hyperdivergent individuals was 1.90 mm (95% CI: 0.82–2.98) for the right condyle and 1.70 mm (95% CI: 0.47–2.93) for the left condyle. Because sex was independently associated with condylar height, this vertical-pattern comparison was also re-analyzed with sex as a covariate (ANCOVA); the effect remained statistically significant for both right condylar height (*p* = 0.0002) and left condylar height (*p* = 0.002), indicating that, unlike the sagittal-pattern findings described above, this association is not attributable to the sex distribution across vertical skeletal pattern groups.

Analysis of condylar morphology and position according to sagittal skeletal patterns revealed no statistically significant differences among the Class I, Class II, and Class III groups. However, significant differences were observed when the condylar position was evaluated according to vertical skeletal patterns. Specifically, among hyperdivergent individuals a centric condylar position was the most common finding (62.2%), whereas a posterior condylar position was relatively more frequent among hypodivergent individuals (42.6%) than in the normodivergent (22.0%) or hyperdivergent (24.3%) groups. The distribution of condylar positions among the vertical skeletal groups was found to be statistically significant (*p* = 0.041) (Table 5). The effect size for this association was Cramér’s V = 0.19 (bootstrap 95% CI: 0.112–0.333), indicating a small-to-moderate association.

Intra-observer agreement for the sagittal skeletal classification, derived from cephalometric tracing, was perfect (κ = 1.000, *p* < 0.001); reliability for the vertical skeletal classification was not separately assessed, as duplicate tracings were not available for this parameter (see Limitations). The intra-observer agreement for categorical variables demonstrated moderate agreement for right condylar morphology (κ = 0.571, *p* < 0.001) and substantial agreement for left condylar morphology (κ = 0.720, *p* < 0.001). Condylar position showed almost perfect agreement on the right side (κ = 0.850, *p* < 0.001) and perfect agreement on the left side (κ = 1.000, *p* < 0.001). For quantitative measurements, intraclass correlation coefficients (two-way random-effects, absolute-agreement, single-measures model, i.e., ICC (2,1)) were excellent for right condylar height (ICC = 0.972, 95% CI: 0.930–0.990, *p* < 0.001), right ramal length (ICC = 0.954, 95% CI: 0.890–0.980, *p* < 0.001), left condylar height (ICC = 0.985, 95% CI: 0.960–0.990, *p* < 0.001), and left ramal length (ICC = 0.943, 95% CI: 0.850–0.980, *p* < 0.001). An initial analysis of the left ramal length data had returned an anomalously low ICC (−0.003, *p* = 0.503); this was traced to a single data-entry error (a misplaced decimal point in one repeat measurement) rather than a genuine reliability problem, and was corrected prior to the analysis reported here. Bland–Altman analysis of the repeat measurements for all four linear parameters showed no systematic trend in the difference across the measurement range, with a small bias (0.14–0.59 mm, greatest for left ramal length) and limits of agreement consistent with the scale of the measurements (approximately ±1–3 mm), supporting the reliability of these measurements (Supplementary Figure S1).

## 4. Discussion

The principal findings of this study indicate a possible association between vertical skeletal pattern and condylar position, observed on the right side only, with the corresponding left-side test being non-significant; given the number of comparisons performed across this study, this side-specific finding should be regarded as hypothesis-generating and requires confirmation in future work. The present study also found that condylar height asymmetry, assessed directly using the Habets index, was significantly greater in individuals with a Class III sagittal pattern. An unadjusted difference in ramus and condylar height was also observed between Class III and Class II individuals, but this difference did not remain significant after adjusting for sex, which differed substantially in distribution between these two groups. The present study evaluated mandibular condylar morphology, position, and linear dimensions including condylar and ramus heights in relation to sagittal and vertical skeletal patterns using CBCT images of adult individuals. Various imaging modalities can be used to assess the TMJ, and the choice of technique depends on whether soft or hard tissues are being examined. Imaging plays an important role in the evaluation of internal TMJ structures. Among available modalities, CBCT enables accurate assessment of osseous components of the TMJ, allowing evaluation of linear and volumetric parameters with relatively low radiation exposure. Furthermore, CBCT provides multiplanar reconstruction without superimposition, magnification, or distortion, thereby improving the precision of condylar morphology and position assessment. Imaging performed in the upright position may also reduce potential diagnostic inaccuracies in condylar position evaluation by maintaining a more natural head posture [29,30,31].

The size and position of the condyle head vary with age and individual characteristics. Morphological changes may also occur due to trauma, syndromes, inflammatory disease, degenerative joint disease, cysts and tumors, metabolic/endocrine disease, developmental abnormalities, malocclusion, and radiotherapy [29,30,32,33]. In this study, medical records of the patients were scanned to eliminate the effects of other factors. To reduce confounding factors, only patients without medical conditions other than malocclusion were included.

Several previous studies have reported similar morphological distributions, indicating that round and convex configurations represent the predominant condylar morphological patterns despite variations in study populations [34,35,36,37]. Previously, Yalçın et al. reported that the predominant form of the right condyle in female patients was convex [31]. However, several other studies have found no significant sex-related differences in condylar morphology [32,34,38].

In the present study, round and convex condylar morphologies were the most frequently observed forms in both females and males, and no statistically significant association was identified between sex and condylar morphology.

In the present study, no significant correlation was found between age and condylar height or ramus height in adult individuals. These findings suggest that, within an adult population, condylar and ramus dimensions may remain relatively stable and are not substantially influenced by age. These results are consistent with a previous research conducted in adult samples, which reported no significant differences in condylar or ramus height according to age [39]. Conversely, studies involving individuals in the active growth phase have demonstrated a positive correlation between age and condylar and ramus height, indicating that both parameters increase during developmental periods [40,41]. On the other hand, studies conducted in skeletally mature individuals have reported that increasing age may be associated with degenerative alterations in the condylar head, which could contribute to a relative reduction in condylar height with advancing age [32,42].

In the present study, sex was found to have a significant effect on condylar and ramus dimensions, with males exhibiting significantly greater right and left condylar height and bilateral ramus height than females. This contrasts with some previous studies that reported no statistically significant sex related differences in condylar or ramus dimensions [38,42,43,44]. The finding of this study is consistent with other studies that have documented sex related differences, with males exhibiting greater ramus height and increased condylar height compared with females [39,45,46,47,48,49].

The literature reports inconsistent findings regarding the relationship between sagittal skeletal pattern and condyle and ramus dimensions. Some studies have found no significant association between sagittal skeletal pattern and condyle or ramus height [11]. In contrast, other studies have demonstrated longer condylar height in Class III individuals [50,51], whereas a shorter condylar height has been reported in Class II individuals [43]. Similarly, a longer ramus height has been observed in Class III patients in some studies [38]. The present study showed an unadjusted difference in right ramus and left condylar height between Class III and Class II individuals, consistent with the findings of Hasebe et al. [52]. However, when sex was accounted for, this difference no longer reached statistical significance, indicating that the apparent sagittal-pattern effect reported here and potentially in similar studies that did not adjust for sex may instead reflect differences in sex composition between the comparison groups rather than a true effect of sagittal skeletal pattern. The varying results reported across studies may be attributed to variations in study design, sample characteristics, age distribution, imaging methods, and measurement techniques. To directly test whether condylar and ramus asymmetry differed by skeletal pattern, the Habets asymmetry index was calculated for each subject. Condylar height asymmetry differed significantly among sagittal skeletal pattern groups, and was significantly greater in Class III individuals than in both Class I and Class II individuals, consistent with reports that asymmetry is more pronounced in individuals with Class III skeletal patterns than in those with other sagittal skeletal patterns. Ramus height asymmetry, in contrast, did not differ significantly by sagittal pattern, but was significantly greater in hyperdivergent than in hypodivergent individuals. Taken together, these findings provide direct statistical support for a tendency toward mandibular asymmetry in individuals with a Class III skeletal pattern [37,39]. The magnitude of the observed condylar height asymmetry exceeded what would be expected from measurement variability alone, based on an empirical error estimate derived from repeat measurements, supporting a genuine biological basis for this finding rather than a measurement artifact.

In the present study, no statistically significant difference was detected between vertical skeletal patterns and ramus height. This finding is consistent with the results reported in earlier studies [11,51]. However, there have been a range of findings regarding the association between vertical skeletal patterns and ramus height. A number of studies have reported significantly greater ramus height in individuals with hypodivergent vertical skeletal patterns compared with individuals with hyperdivergent patterns [14,38,40]. In contrast, Hasebe et al. reported that ramus height was significantly greater in individuals with normodivergent vertical skeletal patterns compared with those in the hyperdivergent group [52]. The observed variations among studies may be attributed to differences in sample composition, age range, imaging methods, and measurement protocols. Overall, the available evidence suggests that, although ramus height may tend to be greater in hypodivergent or normodivergent skeletal patterns, the relationship between vertical skeletal pattern and ramus height remains inconclusive.

In contrast to the present study some previous studies reported no statistically significant differences in condylar height among vertical skeletal patterns [11,14,32,42,50]. The finding of this study is consistent with studies reporting significantly greater condylar height in individuals with hypodivergent patterns than in those with hyperdivergent patterns [14,40,49,53,54].

In the present study, no statistically significant association was detected between sagittal skeletal pattern and mandibular condylar position. This observation is consistent with findings from previous studies, suggesting that the sagittal skeletal pattern alone may not be a definitive factor in determining condylar position [10,13,42,50]. However, the literature presents inconsistent findings regarding this relationship. Vasegh et al. reported that individuals with skeletal Class I pattern exhibited a more anteriorly positioned mandibular condyle compared with those with Class III [43]. However, Bacon et al. reported that the mandibular condyles of Class II individuals were positioned more posteriorly than those of individuals with other sagittal skeletal patterns [55]. The conflicting nature of these findings suggests that the relationship between sagittal skeletal pattern and mandibular condylar position is complex and may be influenced by multiple factors, including occlusal characteristics, craniofacial morphology, and methodological differences among studies.

Condylar height asymmetry, measured directly using the Habets index, was significantly greater in individuals with a Class III sagittal skeletal pattern, supporting a genuine association between sagittal pattern and mandibular asymmetry. An unadjusted difference in ramus and condylar dimensions was also observed between Class III and Class II individuals; however, this difference was no longer significant after accounting for the unequal sex distribution between these groups, and may therefore reflect sex composition rather than a true effect of sagittal skeletal pattern on mandibular dimensions. Despite these findings, no significant relationships were observed between overall condylar position, morphology, and sagittal skeletal patterns, suggesting that condylar characteristics may not be solely determined by sagittal skeletal patterns. Notably, variations in condylar position distribution were observed among vertical skeletal pattern groups, with hyperdivergent individuals more frequently exhibiting a centric position and hypodivergent individuals tending toward a posterior condylar position.

This study has several limitations. Its retrospective, single-center design and the use of an archival CBCT dataset may limit the generalizability of the findings to other populations, and no a priori sample size calculation was performed for the condylar and ramus outcomes reported here. The number of scans excluded at each individual criterion was not tabulated at the time of data collection and could not be reliably reconstructed retrospectively; only the total number of scans screened (3000) and included (132) is available. Most excluded scans were due to tooth loss. All measurements were performed by a single examiner; although intra-observer reliability was assessed and found to be acceptable, the single-examiner design precluded assessment of inter-observer reliability, which is a limitation of the study. Intra-observer reliability was also only moderate for right condylar morphology compared with left condylar morphology; this comparatively lower reliability may partly explain why no significant association was found between condylar morphology and skeletal pattern, and should be considered when interpreting the side-specific findings reported here. A total of 12 chi-square comparisons and 16 ANOVA/Kruskal–Wallis comparisons were performed across the primary categorical and linear outcomes without correction for multiple testing. Several of the significant findings were present on one side but not on the contralateral side (condylar position by vertical pattern; right ramus height and left condylar height by sagittal pattern); given the number of comparisons performed, this pattern is more parsimoniously explained by sampling variability than by genuine side-specific biological effects, and the side-specific significant findings reported here should be interpreted as hypothesis-generating rather than confirmatory. To address mandibular asymmetry directly, the Habets asymmetry index was calculated from paired right and left measurements for each subject; this analysis showed significantly greater condylar height asymmetry in Class III individuals and significantly greater ramus height asymmetry in hyperdivergent individuals. A paired comparison confirmed a small but statistically significant right-greater-than-left offset for ramus height, but not for condylar height (see Results). Although CBCT images were reoriented to the Frankfurt horizontal plane prior to measurement, which reduces the likelihood that this offset reflects sagittal-plane head tilt, this reorientation does not control for transverse positioning asymmetry, which remains a possible contributing factor to the ramus offset alongside true biological asymmetry (e.g., a general tendency toward unilateral, right-dominant mastication). Importantly, the vertical pattern difference in ramus asymmetry index persisted, and was if anything slightly stronger, after centering the data on this offset, indicating that the group difference itself is not simply an artifact of the offset. Sex was the strongest determinant of condylar and ramus dimensions in this dataset; although the sagittal- and vertical-pattern comparisons were each separately adjusted for sex, the potential overlap between the two skeletal classifications was not disentangled in a single combined model. In addition, because ramus height is defined as condylar height plus ramal length (CH + RH), comparisons of these two outcomes are not statistically independent, which should be considered when interpreting parallel findings for both measures. Cephalometric tracing and skeletal-pattern classification were performed by a single orthodontist; intra-observer reliability for the resulting sagittal classification was excellent, but duplicate tracings were not available to assess reliability for the vertical classification specifically, which is an additional limitation given that group allocation underlies every reported comparison. In addition, the sagittal classification rule (ANB and/or Wits) does not pre-specify a hierarchy for the rare cases in which the two indices would indicate different classes, and the number of subjects affected by such disagreement was not separately tracked; nor were exact numerical increased/decreased cut-offs pre-specified for the maxillary height angle and Frankfort mandibular plane angle used in the vertical classification, beyond the approximate reference values stated in the Methods. These classification-protocol details represent a further limitation of the retrospective design. Finally, the cross-sectional nature of the study does not allow causal inferences to be drawn regarding the relationship between skeletal pattern and condylar morphology or position.

## 5. Conclusions

The findings of this study showed that age and sex did not appear to influence condylar morphology or position. Males, however, exhibited significantly greater condylar and ramus height than females. This study directly tested and confirmed mandibular asymmetry using the Habets index: condylar height asymmetry was significantly greater in Class III individuals than in Class I and Class II individuals, while ramus height asymmetry was significantly greater in hyperdivergent individuals than in hypodivergent individuals. In addition, a possible association was observed between condylar position and vertical skeletal patterns on the right side, whereas the corresponding left-side association was not significant: hyperdivergent individuals most frequently presented with a centric condylar position, while hypodivergent individuals showed a relatively more frequent posterior position than the other vertical groups. An unadjusted difference in absolute ramus and condylar height between Class III and Class II individuals appeared attributable to sex composition rather than sagittal skeletal pattern itself.

These findings highlight potential associations between condylar position, ramus–condyle height, condylar and ramus asymmetry, and both vertical and sagittal skeletal patterns. Whether incorporating CBCT based assessment of condylar morphology and position into orthodontic and orthognathic treatment planning improves diagnostic or treatment outcomes was not evaluated in this study and remains a hypothesis for future prospective research.

## Figures and Tables

**Table 1:** Distribution of parameters.

		*n*	%
Sagittal skeletal pattern	Class I	50	37.9
	Class II	44	33.3
	Class III	38	28.8
Vertical skeletal pattern	Normodivergent	41	31.1
	Hypodivergent	54	40.9
	Hyperdivergent	37	28.0
Right condyle morphology	Flat	29	22.0
	Convex	42	31.8
	Angled	25	18.9
	Round	36	27.3
Left condyle morphology	Flat	34	25.8
	Convex	30	22.7
	Angled	28	21.2
	Round	40	30.3
Right condyle position	Anterior	29	22.0
	Centric	62	47.0
	Posterior	41	31.0
Left condyle position	Anterior	36	27.3
	Centric	50	37.9
	Posterior	46	34.8

**Table 2:** Comparison of mandibular condyle position and morphology with sex and age.

		Female *n* (%)	Male *n* (%)	χ^2^	*p* ᵃ	Age (Mean ± SD)	*p* ᵇ
Right Condyle Morphology	Flat	14 (18.4%)	15 (26.8%)	5.312	0.150 ᵃ	28.6 ± 9.4	0.106 ᵇ
	Convex	26 (34.2%)	16 (28.6%)			27.9 ± 7.1	
	Angled	11 (14.5%)	14 (25.0%)			28.3 ± 8.0	
	Round	25 (32.9%)	11 (19.6%)			24.8 ± 7.0	
Left Condyle Morphology	Flat	20 (26.3%)	14 (25.0%)	1.803	0.614 ᵃ	27.7 ± 8.1	0.187 ᵇ
	Convex	16 (21.1%)	14 (25.0%)			28.4 ± 8.5	
	Angled	14 (18.4%)	14 (25.0%)			28.6 ± 7.8	
	Round	26 (34.2%)	14 (25.0%)			25.2 ± 7.0	
Right Condyle Position	Anterior	22 (28.9%)	7 (12.5%)	5.463	0.065 ᵃ	28.2 ± 7.6	0.718 ᵇ
	Centric	31 (40.8%)	31 (55.4%)			27.4 ± 8.9	
	Posterior	23 (30.3%)	18 (32.1%)			26.5 ± 6.2	
Left Condyle Position	Anterior	24 (31.6%)	12 (21.4%)	2.416	0.299 ᵃ	27.6 ± 9.1	0.885 ᵇ
	Centric	25 (32.9%)	25 (44.6%)			27.6 ± 7.7	
	Posterior	27 (35.5%)	19 (33.9%)			26.7 ± 7.2	

^a^ Chi-square test, ^b^ Kruskal–Wallis test, SD: standard deviation.

**Table 3:** Comparison of mandibular condyle and ramus height with sex and age.

	Female (Mean ± SD)	Male (Mean ± SD)	Test Statistic (t/U)	*p*	Age	
					Spearman’s r	*p*
Right Condyle Height	7.9 ± 1.9	9.9 ± 2.7	−4.917	<0.001 *ᵃ	0.110	0.211 ᶜ
Left Condyle Height	7.7 ± 2.0	10.0 ± 3.1	3127.0	<0.001 *ᵇ	0.071	0.417 ᶜ
Right Ramus Height	48.3 ± 4.0	52.7 ± 4.1	−6.125	<0.001 *ᵃ	0.044	0.614 ᶜ
Left Ramus Height	47.4 ± 4.2	51.9 ± 5.4	−5.475	<0.001 *ᵃ	0.152	0.081 ᶜ

^a^ Student’s *t*-test, ^b^ Mann–Whitney U test, ^c^ Spearman test, SD: standard deviation, *p* * < 0.05.

**Table 4:** Condyle–ramus height according to condyle position, sagittal and vertical skeletal patterns.

		Right Condyle Height (Mean ± SD)	Right Ramus Height (Mean ± SD)	Left Condyle Height (Mean ± SD)	Left Ramus Height (Mean ± SD)
Sagittal skeletal pattern	Skeletal Class I	9.0 ± 2.15	50.40 ± 4.89 †‡	8.97 ± 2.18 †‡	49.72 ± 5.04
	Skeletal Class II	8.07 ± 2.57	48.39 ± 3.87 †	7.84 ± 2.65 †	47.9 ± 4.56
	Skeletal Class III	9.18 ± 2.62	52.03 ± 4.38 ‡	9.22 ± 3.35 ‡	50.44 ± 5.85
	*p*	0.082 ᵃ	0.001 *ᵃ	0.046 *ᵃ	0.069 ᵃ
Right condyle position	Anterior	9.11 ± 2.15	49.47 ± 4.39	—	—
	Centric	8.62 ± 2.66	50.6 ± 5.14	—	—
	Posterior	8.67 ± 2.38	50.1 ± 3.95	—	—
	*p*	0.656 ᵃ	0.549 ᵃ	—	—
Left condyle position	Anterior	—	—	9.59 ± 3.33	50.44 ± 5.89
	Centric	—	—	8.33 ± 2.39	49.21 ± 5.12
	Posterior	—	—	8.31 ± 2.51	48.57 ± 4.66
	*p*	—	—	0.062 ᵃ	0.270 ᵃ
Vertical skeletal pattern	Normodivergent	8.4 ± 1.9	50.1 ± 4.2	8.1 ± 2.0	49.1 ± 4.7
	Hypodivergent	9.7 ± 2.9	50.7 ± 4.9	9.6 ± 3.1	50.1 ± 5.4
	Hyperdivergent	7.8 ± 1.9	49.7 ± 4.7	7.9 ± 2.6	48.5 ± 5.3
	***p***	0.004 *ᵇ	0.599 ᵃ	0.011 *ᵇ	0.322 ᵇ

^a^ One-way Anova test, ^b^ Kruskal–Wallis test, p * < 0.05. †, ‡ Homogeneous subsets from post hoc pairwise comparisons (values sharing a symbol are not significantly different from one another).

**Table 5:** Condyle position and morphology according to sagittal and vertical skeletal patterns. Values are expressed as row percentages (i.e., the percentage of each skeletal-pattern group falling into each position or morphology category).

	Right Condyle Position			Left Condyle Position			Right Condyle Morphology				Left Condyle Morphology			
	A	C	P	A	C	P	F	Co	An	R	F	Co	An	R
**Class I**	15 (30.0%)	17 (34.0%)	18 (36.0%)	14 (28.0%)	14 (28.0%)	22 (44.0%)	10 (20.0%)	15 (30.0%)	9 (18.0%)	16 (32.0%)	11 (22.0%)	13 (26.0%)	13 (26.0%)	13 (26.0%)
**Class II**	9 (20.5%)	23 (52.3%)	12 (27.3%)	10 (22.7%)	21 (47.7%)	13 (29.5%)	14 (31.8%)	13 (29.5%)	6 (13.6%)	11 (25.0%)	12 (27.3%)	11 (25.0%)	7 (15.9%)	14 (31.8%)
**Class III**	5 (13.2%)	22 (57.9%)	11 (28.9%)	12 (31.6%)	15 (39.5%)	11 (28.9%)	5 (13.2%)	14 (36.8%)	10 (26.3%)	9 (23.7%)	11 (28.9%)	6 (15.8%)	8 (21.1%)	13 (34.2%)
***p***	0.163			0.290			0.397				0.776			
**Normodivergent**	13 (31.7%)	19 (46.3%)	9 (22.0%)	12 (29.3%)	16 (39.0%)	13 (31.7%)	6 (14.6%)	13 (31.7%)	10 (24.4%)	12 (29.3%)	11 (26.8%)	8 (19.5%)	12 (29.3%)	10 (24.4%)
**Hypodivergent**	11 (20.4%)	20 (37.0%)	23 (42.6%)	14 (25.9%)	17 (31.5%)	23 (42.6%)	13 (24.1%)	19 (35.2%)	6 (11.1%)	16 (29.6%)	9 (16.7%)	15 (27.8%)	8 (14.8%)	22 (40.7%)
**Hyperdivergent**	5 (13.5%)	23 (62.2%)	9 (24.3%)	10 (27.0%)	17 (45.9%)	10 (27.0%)	10 (27.0%)	10 (27.0%)	9 (24.3%)	8 (21.6%)	14 (37.8%)	7 (18.9%)	8 (21.6%)	8 (21.6%)
***p***	**0.041** *			0.554			0.474				0.105			

Chi-square test p* < 0.05. A: Anterior, C: Centric, P: Posterior, F: Flat, Co: Convex, An: Angled, R: Round.

## Data Availability

The data presented in this study are available on request from the corresponding author due to institutional restrictions on patient imaging data.

## Supplementary Materials

The following supporting information can be downloaded at: https://www.mdpi.com/article/10.3390/jcm15186991/s1.
