# Supplementary material for: Assessment of the Position and Height of the Mandibular Condyle in Different Skeletal Patterns Using CBCT
Source: J Clin Med. 2026 Sep 9;15(18):6991. doi: 10.3390/jcm15186991 (PMC13607097; doi:10.3390/jcm15186991)
Supplement: Supplementary file 1 [file jcm-15-06991-s001.zip › jcm-4481921-supplementary.pdf]

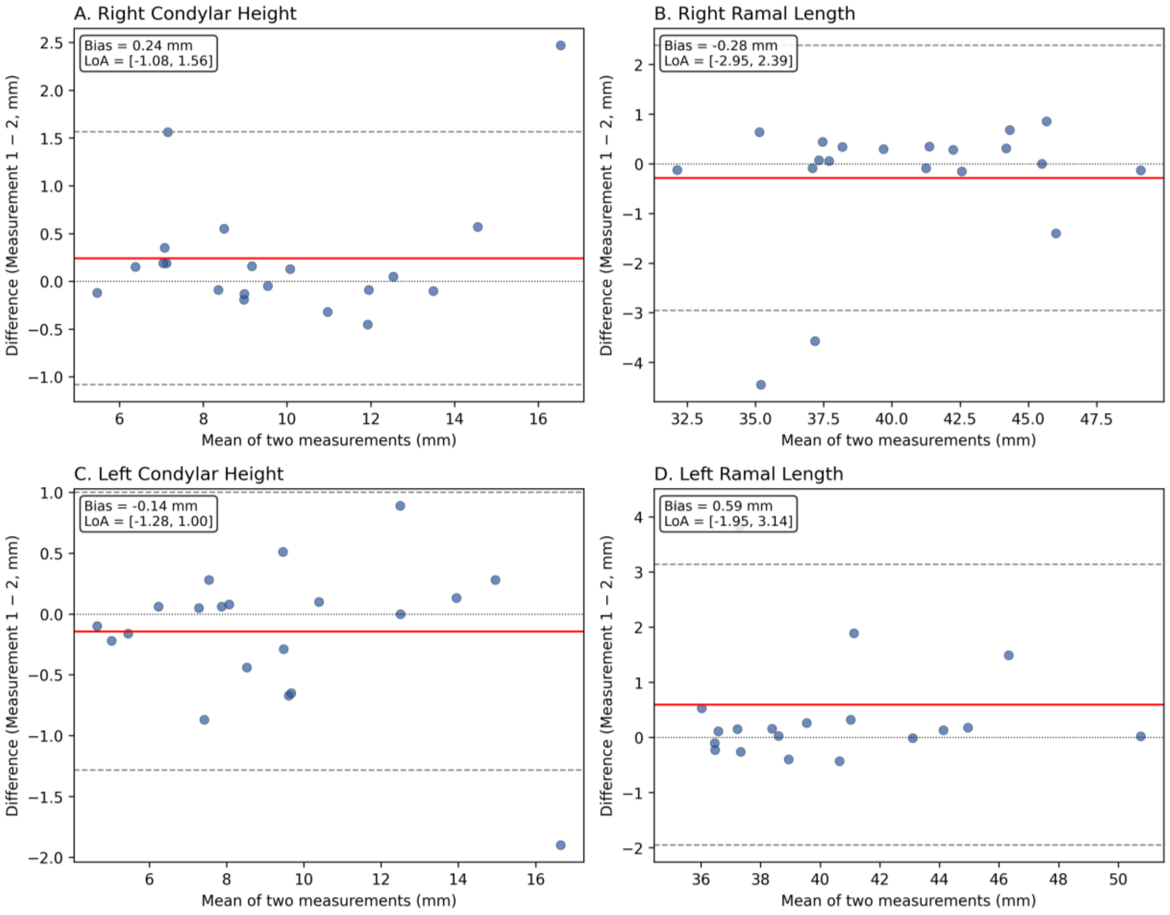

Figure S1. Bland-Atman plots for intra-observer reliability of linear measurements (n = 20 repeat pairs).
